# Influences of Selenium-Enriched Yeast on Growth Performance, Immune Function, and Antioxidant Capacity in Weaned Pigs Exposure to Oxidative Stress

**DOI:** 10.1155/2021/5533210

**Published:** 2021-03-27

**Authors:** Lei Liu, Daiwen Chen, Bing Yu, Yuheng Luo, Zhiqing Huang, Ping Zheng, Xiangbing Mao, Jie Yu, Junqiu Luo, Hui Yan, Jun He

**Affiliations:** ^1^Institute of Animal Nutrition, Sichuan Agricultural University, Sichuan Province, Chengdu 611130, China; ^2^Key Laboratory of Animal Disease-Resistant Nutrition, Sichuan Province, Chengdu 611130, China

## Abstract

This study elucidated the function role of dietary selenium-enriched yeast (SeY) supplementation on growth performance, immune function, and antioxidant capacity in weaned pigs exposure to oxidative stress. Thirty-two similarity weight pigs were randomly divided into four treatments: (1) nonchallenged control, (2) control+SeY, (3) control+diquat, and (4) control+SeY+diquat. The period of experiment was 21 days; on day 16, pigs were injected with diquat or sterile saline. Results revealed that oxidative stress was notably detrimental to the growth performance of piglets, but SeY supplementation ameliorated this phenomenon, which might be regarding the increasing of body antioxidant capacity and immune functions. In details, SeY supplementation improved the digestibility of crude protein (CP), ash, and gross energy (GE). Moreover, the serum concentrations of proinflammatory cytokines (TNF-*α*, IL-1*β*, and IL-6), glutamic-pyruvic transaminase(GPT), and glutamic-oxaloacetic transaminase (GOT) were reduced via SeY supplemented, and serum concentrations of immunoglobulins A (IgA), IgG, and activities of antioxidant enzymes such as the superoxide dismutase (SOD), catalase (CAT) ,and glutathione peroxidase (GSH-Px) were improved in the diquat-challenged pigs (*P* < 0.05). In addition, SeY supplementation acutely enhanced the activities of these antioxidant enzymes in the liver and thymus upon diquat challenge, which involved with the upregulation of the critical genes related antioxidant signaling such as the nuclear factor erythroid-derived 2-related factor 2 (Nrf-2) and heme oxygenase-1 (HO-1) (*P* < 0.05). Importantly, we also found that SeY supplementation apparently reduced the malondialdehyde (MDA) concentrations in the liver, thymus, and serum (*P* < 0.05). Specifically, the expression levels of TNF-*α*, IL-6, IL-1*β*, Toll-like receptor 4 (TLR-4), and nuclear factor-*κ*B (NF-*κ*B) in the liver and thymus were downregulated by SeY upon diquat challenge. These results suggested that SeY can attenuate oxidative stress-induced growth retardation, which was associated with elevating body antioxidant capacity, immune functions, and suppressed inflammatory response.

## 1. Introduction

Recent studies have shown that neonatal animals such as the weaning pigs (approximately 3-5 weeks of age) are more prone to gastrointestinal diseases, infections, and diarrhea because of incomplete development of immune and digestive systems [1]. In addition, weaning can cause a variety of oxidative stress-induced injuries, including growth retardation, disease, and even death, resulting in considerable economic losses [2]. Under normal circumstances, ROS produced in the body participates a variety of biological events including signal transduction, gene expression, and activation of receptors [3]. Excessive production of oxidative radicals is generally neutralized or eliminated by the antioxidant system including nonenzymatic components, such as glutathione, selenium, vitamin E, and vitamin C, and a series of antioxidant enzymes, such as SOD, CAT, and GSH-Px [4, 5]. Previous study indicated that overproduction of ROS can bring the damage to DNA, proteins, and lipids and further provoke the production of reactive species such as MDA and 4-hydroxynonenal [6, 7]. As a result, oxidative stress is harmful and causes irreversible damage to the cell structure and function, leading to cell death through the process of necrosis and apoptosis (Hetz & Claudio, 2007).

Selenium is an essential trace element in the animal body [8], which exerts various functions in improving antioxidant capacity, immunity, growth, and meat quality [9]. Selenium plays an important role in pig nutrition via participating in selenoprotein synthesis, which is central for the antioxidant system regulation in the body [10]. As compared to inorganic selenium (i.e., sodium selenite and sodium selenate), organic selenium (i.e., selenium methionine and selenium-enriched yeast) has higher absorption and utilization rates [11]. Previous study indicated that selenium-enriched yeast can relieve various oxidative stress-induced damages through improving the body antioxidant enzymes activities [12]. However, our understanding of the precise growth-promoting function of SeY remains indistinct, especially weaned pigs in the oxidative stress. Thus, to clarify the role of SeY, we investigated the effect of dietary SeY supplementation on growth performance, immune function, and antioxidant capacity in weaned pigs upon oxidative stress. Meanwhile, the mechanisms for the SeY-modulated immune and antioxidative functions have also been explored. This study could also provide novel insights into the application of SeY for the livestock industry.

## 2. Materials and Methods

### 2.1. Animal Trial

Thirty-two similarity weight (7.30 ± 0.14 kg) pigs were purchased from a commercial farm. The weaned pigs with the same breed (DLY) and gender and nearly the same initial body weight (age) were used in this study and randomly divided into four treatments consisting of nonchallenged pigs (CON, fed with basal diet), diquat-challenged pigs (DT, fed with basal diet), and SeY-treated pigs (fed with basal diet containing 250 mg/kg SeY) challenged by sterile saline (SSY) or diquat (DSY). Diquat came from Shanghai Yuanye Biotechnology CO., Ltd., average molecular weight 362.06. and purity of 99%, then prepared into 10 mg/kg diquat solution with sterilized physiological saline and stored at 4-8°C (Shanghai, China). The SeY was purchased from Sichuan Junzheng Biofeed Co., Ltd. (Organic Selenium ≥95%). We used the recommendations of the National Research Council in 2012 [13] to formulate the nutritional requirements of basal diet (Table 1). Pigs were kept in metabolism cages individually and fresh water and feed available. The trial conducted for 21 d. The oxidative stress model was established by using diquat. On 16 d, pigs received intraperitoneally injection of sterile saline (0.9%) or diquat (10 mg/kg BW).

### 2.2. Sample Collection and Preparation

To begin with the trial, about 150 g of each treated diet samples was collected by the quartering method, the sterile plastic bags containing feed samples were vacuum-extracted, and the source information was marked and stored at -20°C for chemical analysis. The digestion test was carried out for all piglets on days 12-15 of the experiment, and 10 mL of a 10% H_2_SO_4_ solution and 0.5 mL of toluene were added to 100 g fresh feces for preservative and nitrogen fixation. An appropriate amount of fully mixed feces was taken and placed in the sample plate, dried at 60-65°C to a constant weight, dried and crushed, screened for 40 mesh, and stored at -20°C for subsequent nutrient analysis. At the end of the experiment (morning of day 22), all pigs were weighed on an empty stomach, and blood was collected from the anterior vena cava. The samples were placed at room temperature for 30 min and centrifuged at 3000 r/min for 15 min. The supernatant was collected and stored at -20°C for the detection of various physiological and biochemical indexes in the serum. After blood collection, the sodium pentobarbital was used to euthanize all the pigs through intravenous injection and slaughtered by exsanguination protocols. The liver, spleen, kidney, and thymus were weighted, collected, and snap frozen in liquid N_2_ and then stored at −80°C until analysis.

### 2.3. Measurement of Growth Performance

Daily feed intake of each pig was accurately recorded throughout the experiment. Fasting weightings were performed on the morning of days 1 and 22, respectively. Average daily gain (ADG), average daily feed intake (ADFI), and feed/gain (F/G) of each pig from stages 1 to 21 were recorded.

### 2.4. Determination of the Apparent Total Tract Digestibility

Determination of apparent total gastrointestinal digestibility (ATTD) calculated by acid insoluble ash (AIA) [14]. Chinese National Standard (GB/T23742) was applied to measure the AIA in diets and feces samples. DM, CP, EE, and ash were determined in the samples and fecal samples. Also, adiabatic bomb calorimeter (LECO, St. Joseph, Michigan, USA) was utilized to calculate the GE content of diets and fecal samples. Finally, the ATTD was carried out by (100 − A1F2/A2F1 × 100), where A1 serves as the AIA content of the diet, A2 acts as the AIA content of feces, F1 regards as the nutrient content of the diet, and F2 represents the nutrient content of feces.

### 2.5. Measurement of the Viscera Indexes

Liver, spleen, kidney, and thymus were separated and weighed. The relative organ weight was calculated according to the ratio of the organ weight to the body weight [15].

### 2.6. Measurements of Serum Immunoglobulin and Cytokine Concentrations

Serum contents of TNF-*α*, IL-1*β*, IL-6, TP, and immunoglobulin (Ig) consisting of IgA and IgG were determined referred to the procedures described by the corresponding kit manufacturer using commercially available swine enzyme-linked immunosorbent assay (ELISA) kits (Jiangsu Jingmei Biotechnology Co., Ltd., Yancheng, China).

### 2.7. Antioxidant Enzymes and MDA Concentration in the Serum, Thymus, and Liver

The MDA content and the activities of several enzymatic as well as nonenzymatic antioxidants consists of GSH-P_X_, CAT, SOD, and T-AOC were tested in the serum, thymus, and liver. All antioxidant-related indices were detected using the corresponding diagnostic kits obtained from Nanjing Jian Cheng Bioengineering Institute (Nanjing, China) that were used according to the manufacturer's instructions.

### 2.8. Measurement of Serum GOT and GPT

The activities of GOT and GPT were measured using commercial kits (Nanjing Jiancheng Biological Product, Nanjing, China) according to the manufacturer's recommendations.

### 2.9. Gene mRNA Expression Analysis by Real-Time PCR

TRIzol Reagent (TaKaTa, Dalian, China) was used to extract the total RNA from the liver and thymus on the basis of the manufacturer's instructions. Spectrophotometer (Beckman Coulter DU800; Beckman Coulter Inc.) was applied to measure the concentration and purity of RNA at 260 and 280 nm to ensure the ratios of absorption (OD260/OD280 nm) various between 1.8 and 2.0 for all samples. The integrity of RNA was verified by formaldehyde gel electrophoresis. Reverse transcription of each sample was conducted by PrimeScript RT reagent kit with gDNA Eraser (TaKaRa, Dalian, China) following the manufacturer's instructions. The primers were synthesized commercially by Life Technologies Limited and exhibited in Table 2.

The analysis of the expression abundance of TNF-*α*, IL-6, IL-1*β*, TLR4, NF-*κ*B, Nrf-2, and HO-1 was calculated by quantitative real-time PCR in the liver and thymus using SYBR Premix Ex Taq II (Tli RnaseH Plus) reagents (TaKaRa, Dalian, China) and the CFX96 Real-Time PCR Detection System (Bio-Rad Laboratories, Richmond, CA). The *β*-actin gene was chosen as the reference gene to normalize the mRNA expression of target genes. The relative expression ratio of target genes relative to the reference gene was calculated using the 2^−*ΔΔ*CT^ method [16]. Each sample was simultaneously performed on the same PCR plate.

### 2.10. Statistical Analysis

Data were carried out as a 2 × 2 factorial arrangement by ANOVA through the general linear model procedure of SPSS 24.0 (SPSS Inc., Chicago). The statistical model contains the effects of diquat (challenged or unchallenged), SeY (supplemented or not supplemented), and their interaction. Data were exhibited as means and stand errors. Differences were regarded as significant when *P* < 0.05. Meanwhile, *P* < 0.10 is discussed as trends. Variable means for treatments showing significant differences in the ANOVA were separated by Tukey's multiple range test (*P* < 0.05).

## 3. Results

### 3.1. Growth Performance

As showed in Table 3, the ADG and ADFI were cut down in the DT group than the CON group (*P* < 0.05). Dietary SeY supplementation significantly promoted ADG, ADFI, and the feed efficiency in the diquat-challenged pigs (*P* < 0.05).

### 3.2. Effect of SeY on Nutrient Digestibility in Weaned Pigs

SeY supplementation had no effect on the apparent digestibility of DM and EE (Table 4). The digestibility of CP and ash was significant higher in SSY group than the CON group (*P* < 0.05). Interestingly, SeY supplementation significantly elevated the digestibility of GE and ash in the DSY group than the DT group (*P* < 0.05).

### 3.3. Effect of SeY on the Viscera Indexes in Weaned Pigs upon Oxidative Stress

As showed in Table 5, dietary SeY supplementation had no effect on the liver, spleen, kidney, and thymus index under normal condition (*P* > 0.05). However, the indexes of the liver and kidney in the DSY group were significantly higher than those in the DT group (*P* < 0.05). There was no effect on the spleen index under both conditions (*P* > 0.05).

### 3.4. Effect of SeY on Serum GPT and GOT in Weaned Pigs upon Oxidative Stress

**Figure 1:**
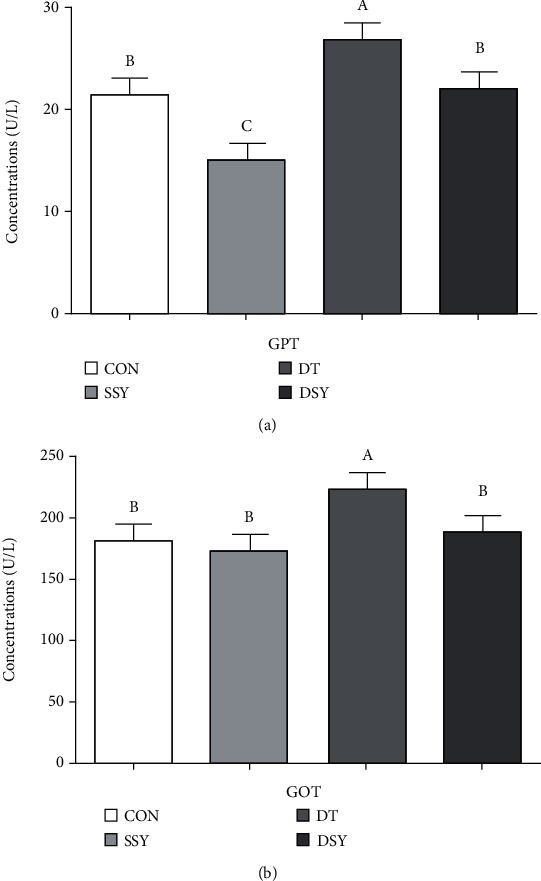
Effect of SeY on serum GPT and GOT in weaned pigs upon oxidative stress. (a)–(c) Mean values with different letters on vertical bars indicate significant differences (*P* < 0.05). CON, pigs were fed with basal diet and challenged by sterile saline, SSY: pigs were fed with SeY-containing diet and challenged by sterile saline, DT: pigs were fed with basal diet and challenged by diquat, and DSY: pigs were fed with SeY-containing diet and challenged by diquat. GPT: glutamic-pyruvic transaminase; GOT: glutamic-oxaloacetic transaminase.

As shown in Figure 1, compared to the CON group, the GPT content in the serum was significantly lowered in the SSY group (*P* < 0.05). However, the GOT and GPT levels were significantly reduced in the DSY group compared to the DT group (*P* < 0.05).

### 3.5. Effect of SeY on Serum Immunoglobulin and Cytokine Concentrations in Weaned Pigs upon Oxidative Stress

Dietary SeY supplementation significantly amplified the serum TP, IgG, and IgA levels and inhibited the serum IL-1*β* concentration in weaned piglets without diquat administration (*P* < 0.05, Table 6); however, dietary SeY supplementation also reduced the TNF-*α*, IL-1*β*, and IL-6 (*P* < 0.05) content in piglets when piglets were subjected to the diquat injection. In addition, the contents of TP, IgG, and IgA in the DSY group were higher than those in the DT group.

### 3.6. Effect of SeY on Antioxidant Capacity and MDA Content in the Serum

The data for antioxidant capacity parameters in serum are presented in Table 7. Compared to the CON group, higher serum content of the MDA was observed in the DT group (*P* < 0.05). However, SeY supplementation not only brought down the MDA content but also enhanced the GSH-Px, CAT, and SOD activities in the serum (*P* < 0.05). Compared to the DT group, the T-AOC was extremely raised in the DSY group (*P* < 0.05).

### 3.7. Effect of SeY on Antioxidant Capacity and MDA Content in the Liver and Thymus

As shown in Table 8, compared to the CON group, the MDA content in the liver and thymus was significantly reduced in the SSY group (*P* < 0.05), and the CAT and SOD activities were significantly reduced in the DT group (*P* < 0.05). However, the T-AOC, CAT, and SOD activities in the liver and the GSH-Px, SOD, and CAT activities and T-AOC in the thymus were significantly elevated in the DSY group compared with the DT group (*P* < 0.05).

### 3.8. Effect of SeY on Expressions of Critical Genes Related to the Inflammatory Response

**Figure 2:**
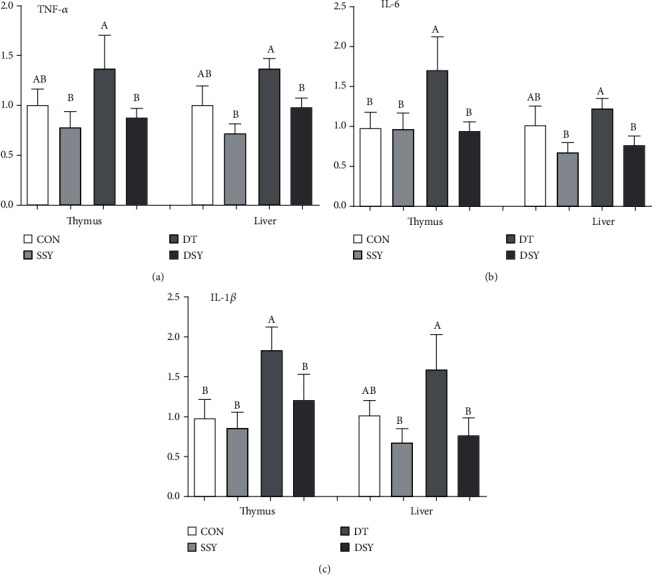
Relative expression levels of critical genes involved in the inflammatory response. (a)–(c) Mean values with different letters on vertical bars indicate significant differences (*P* < 0.05). CON, pigs were fed with basal diet and challenged by sterile saline, SSY: pigs were fed with SeY-containing diet and challenged by sterile saline, DT: pigs were fed with basal diet and challenged by diquat, and DSY: pigs were fed with SeY-containing diet and challenged by diquat. TNF-*α* :tumor necrosis factor-*α*; IL-6 interleukin-6; IL-1*β*: interleukin-1*β*.

**Figure 3:**
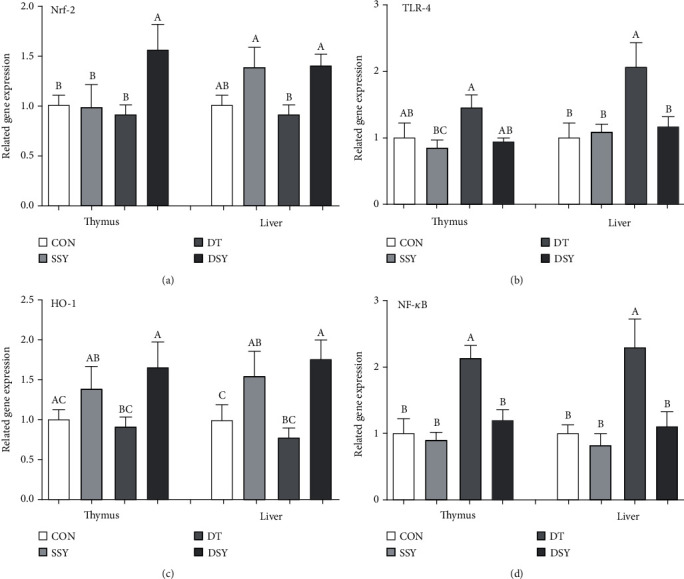
Relative expression levels of critical genes involved in the inflammatory response. (a)–(c) Mean values with different letters on vertical bars indicate significant differences (*P* < 0.05). CON: pigs were fed with basal diet and challenged by sterile saline, SSY: pigs were fed with SeY-containing diet and challenged by sterile saline, DT: pigs were fed with basal diet and challenged by diquat, and DSY: pigs were fed with SeY-containing diet and challenged by diquat. TLR-4: Toll-like receptor 4; NF-*κ*B: nuclear factor-*κ*B; Nrf-2: nuclear factor erythroid-derived 2-related factor 2; HO-1: heme oxygenase-1.

As shown in Figure 2, compared to the CON group, the expression abundance of IL-6 and IL-1*β* was upregulated in the DT group of the liver and thymus (*P* < 0.05); however, their expressions were declined in the DSY group. Lower expression levels of TNF-*α* in the liver and thymus were found in the DSY group compared with the DT group (*P* < 0.05). Meanwhile, the expression levels TLR-4 and NF-*κ*B in the liver were upregulated in the DT group compared with the CON group (Figure 3); on the contrary, their expressions were downregulated in the DSY group. The expression abundance of Nrf-2 and HO-1 in the liver and thymus was upper in the DSY group than the DT group (*P* < 0.05). However, no effect was occurred to the expression of TNF-*α*, IL-6, and IL-1*β*, TLR4, NF-*κ*B, Nrf-2, and HO-1 in the SSY group compared with the CON group (*P* > 0.05).

## 4. Discussion

To evaluate whether SeY supplementation could alleviate oxidative stress injury through improving body antioxidative capacity and immune functions roles in weaned pigs, we established a model for inducing oxidative stress in pigs by using diquat injection [17]. Multiple lines of evidence show that diquat-induced oxidative stress has been used commonly as an experimental animal model in which to analyze the mechanism of chemical agent-induced acute oxidative injury [18]. Vomiting and anorexia were initially occurred to all piglets with diquat challenge. In parallel with our study, diquat injected was adverse to the growth performance indicating conspicuously dropped down the ADG and ADFI, which are consistent with previous observations showing that the performance of weaned pigs are negatively affected by diquat challenge [19]. Meanwhile, dietary SeY supplementation significantly promoted ADG, ADFI, feed efficiency, and apparent nutrient digestibility in pigs. It is suggested that SeY exerted a potentially crucial role in inhibiting adverse reactions in weaned piglets exposure to the oxidative stress. This result also is in agreement with former outcomes that SeY supplementation can boost the growth performance and antioxidant capacity of animals under oxidative stress [20].

Nutrition and management at the weaning phase are crucial to the growth and development of piglets. Additionally, in this phase, piglets are more susceptible to multifarious stresses (i.e., oxidative stress), and oxidative stress exploits full advantages to the pathophysiology of numerous cardiovascular diseases (i.e., endothelial dysfunction, atherosclerosis, and ischemia–reperfusion injury). Exceeding accumulation levels of ROS in the animal body—such as H_2_O_2_, O_2_^−^, and OH^−^—seriously undermine the function of proteins, DNA, and lipids, which lead to tissue injury and organ dysfunction, causing considerable economic losses to the pig industry [21]. The homeostasis of the ROS is maintained in vivo associating with signal transduction, gene expression, and receptor activation [22]. Nevertheless, excessive accumulation of ROS is usually removed promptly by the antioxidant system, including nonenzymatic components, such as vitamins E and C, and a range of antioxidant enzymes consisting of SOD, CAT, and GSH-Px. They are considered to be the principal line of the antioxidant enzyme system to boycott ROS production subjected to oxidative stress. SOD is distributed in plenty of tissues and organisms, which exert the protective function role of cells injury caused by O_2_^–^. CAT and GSH-Px can serve as catalysts to convert H_2_O_2_ into nontoxic H_2_O. Furthermore, T-AOC is a participant in the nonenzymatic antioxidant defense systems [23]. The SeY is reported to be a particularly attractive antioxidant due to its stability in the presence of strong function in reducing the production of lipid peroxidation, eliminating ROS, relieving DNA oxidative injury, and preventing cell apoptosis. Our experiment found that dietary SeY supplementation significantly increased the activities of T-AOC, SOD, CAT, and GSH-Px in the serum, liver, and thymus, suggesting that dietary SeY supplementation played protective role on diquat-induced oxidative stress of weaned piglets by enhancing the antioxidant function.

Cellular biomembranes are one of the main targets subjected to the attack of ROS, which in turn induces lipid peroxidation, resulting in MDA production. MDA can interact with biomolecules result in cytotoxicity and genotoxicity. Collectively, the detection of the MDA content is usually used as a key label for oxidative stress in organisms [24]. We found that dietary supplementation of SeY significantly reduced the content of the MDA in the serum, thymus, and liver, declaring that SeY supplementation suppressed lipid peroxidation. Our results are in line with the former studies that SeY supplementation could reduce oxidative stress and has the potential effects on ameliorating the deleterious impacts of oxidative stress injury. Organ index reflects the development and functional status of the organ. In the present study, the liver and kidney index was significantly improved in the DSY group compared with the DT group, indicating that SeY had beneficial effects on attenuating the tissue injury of weaned pigs undergoing oxidative stress.

GOT and GPT are hepatic intracellular enzymes, which principally work in the synthesis and metabolism of nonessential amino acids. Excessive serum elevation of these two enzymes revealed leakage of damaged liver cells and is regarded as sensitive indexes of liver injury [25]. In the present study, the serum activities of GOT and GPT were apparently reduced in the DSY group than the DT group, indicating that SeY had favorable effect in improving the liver function upon diquat injection. These results also got agreement with former findings [26].

Linkages between oxidative stress and inflammation are closely correlated [27]. In addition, oxidative stress disrupts the body's redox balance, which also leads to systemic inflammation. Systemic responses may be triggered by the overexpression of proinflammatory (TNF-ɑ, IL-6, and IL-1*β*) in the body, resulting in cell damage [28]. Both IgA and IgG are crucial markers of the body's immune capacity and figure a profoundly essential role in the body's response to a series of pathogens [29, 30]. Total serum protein is a complex mixture of proteins, consisting of ALB and globulin, which on behalf of the nutritional levels of dietary protein available to the animal as well as the degree of digestion and absorption [31]. Previous studies demonstrated that dietary SeY supplementation enhances the immune response in early weaned piglets and mice as well as the human by modulating the production of cytokines and antibodies through enhancing B lymphocytes and their functional activity [32, 33]. In this study, we observed that SeY prominently elevated the contents of serum TP, IgA, and IgG and downregulated the content of the IL-1*β*, IL-6, and TNF-*α* in piglets exposed to oxidative stress. These findings were in accordance with the reports. On the basis of these results, we can come to a conclusion that dietary supplementation of SeY showed great advantages over alleviating body injury of diquat-challenged pigs. It is speculated that the protective effect of SeY may be related to the decrease of serum proinflammatory cytokines and the advancement immunoglobulin level.

To elucidate the molecular mechanisms by which SeY supplementation might attenuate thymus and liver antioxidative function and inflammatory response under oxidative stress, we determined several vital genes associating with the antioxidative function and inflammatory response. TLR4 is a typical member in the TLR family and widely distributed on various types of cells [27, 34]. Activation of TLR4 accompanies with initiating the activation of NF-*κ*B signal pathway and then expands the expression levels of omnifarious inflammatory cytokines' genes, including IL-6, TNF-*α*, and IL-1*β* [35]. In the present study, we have observed that the expression levels of TLR4, NF-*κ*B, TNF-*α*, IL-6, and IL-1*β* in the liver and thymus were lower in the DSY group than the DT group. Therefore, the protective effect of SeY on oxidative stress injury in the liver and thymus may be related to the downregulation mRNA expression of TLR4 signaling-related genes. Together speaking, our results are also consistent with reports that SeY improves hepatic protection by regulating proinflammatory cytokines and antioxidant capacity in broilers under heat stress conditions [36].

Nrf-2 is an essential redox-sensitive transcription component that has been involved in cellular responses to oxidative stress, which coordinates the expression of multiple genes such as antioxidant and detoxification via a motion-promoting sequence named antioxidant response element (ARE), which synergically forms a pleiotropic cellular defense that eliminates ROS, detoxifies electrophiles and xenobiotics, and maintains the reductive potential in the cell [37]. HO-1 is an important antioxidant enzyme that plays a key role in endogenous and exogenous cell protection against harmful stimuli. On the one hand, its antioxidant function is related to the prevention of free heme from participating in the oxidative reaction; on the other hand, HO-1 and its enzymolysis products including bilirubin and CO exert a key role in antioxidant, anti-inflammatory and inhibiting cell apoptosis. Accumulating evidence suggesting that the Nrf-2/HO-1 pathway is one of the most important endogenous protective systems, which is widely involved in the oxidative stress injury of the heart, brain, liver, kidney, and nervous system [38]. In the present study, we observed that the expression abundance of the Nrf-2 and HO-1 was elevated in the DSY group compared with the DT group in the liver and thymus subjected to oxidative stress. The result is accompanied with the measurements of the antioxidative enzymes in the liver and thymus. Both results indicated that the SeY could act as a potential therapeutic antioxidant agent to relieve the tissue injury induced by diquat injection.

## 5. Conclusions

SeY plays a key role in antioxidation and anti-inflammation. In conclusion, dietary SeY supplementation alleviates the negative effects of diquat injection on growth performance, inflammatory response, and organ injury in piglets via improving antioxidative activity. These results would provide scientific evidences for the development of antioxidant therapies and anti-inflammatory drugs in the future as well as the application of SeY in piglets.

## Figures and Tables

**Table 1:** Composition and nutrient levels of experimental diets.

Ingredients	%	Nutrient level	Contents
Corn	28.31	Digestible energy (calculated, MJ/kg)	14.78
Extruded corn	24.87	Crude protein(%)	19.68
Soybean meal	8.50	Calcium(%)	0.81
Extruded full-fat soybean	10.30	Available phosphorus (%)	0.55
Fish meal	4.20	Lysine	1.35
Whey powder	7.00	Methionine	0.42
Soybean protein concentrate	8.00	Methionine+cysteine	0.60
Soybean oil	2.00	Threonine	0.79
Sucrose	4.00	Tryptophan	0.22
Limestone	0.90		
Dicalcium phosphate	0.50		
NaCl	0.30		
L-Lysine$HCl (78%)	0.47		
DL-Methionine	0.15		
L-Threonine (98.5%)	0.13		
Tryptophan (98%)	0.03		
Chloride choline	0.10		
Vitamin premix^a^	0.04		
Mineral premix^b^	0.20		
Total	100		

^a^The vitamin premix provided the following per kg of diet: 9000 IU of VA, 3000 IU of VD 3, 20 IU of VE, 3 mg of VK 3, 1.50 mg of VB1, 4 mg of VB 2, 3 mg of VB6, 0.02 mg of VB12, 30 mg of niacin, 15 mg of pantothenic acid, 0.75 mg of folic acid, and 0.10 mg of biotin. The premix provided the following per kg of diets: 75 mg of Fe, 150 mg of Cu, 75 mg of Zn, 60 mg of Mn, and 0.35 mg of I.

**Table 2:** Primers sequences used for quantitative RT-PCR.

Gene	Primer sequences (5′-3′)	Product length, bp
Nrf2	F: GCCCCTGGAAGCGTTAAAC	67
	R: GGACTGTATCCCCAGAAGGTTGT	
HO-1	F: CGCTCCCGAATGAACAC	112
	R: GCTCCTGCACCTCCTC	
TNF-*α*	F: TCTCATGCACCACCATCAAGGACT	178
	R: ACCACTCTCCCTTTGCAGAACTCA	
IL-1*β*	F: ACCTGTGTCTTTCCCGTGG	92
	R: TCATCTCGGAGCCTGTAGTG	
IL-6	F: ATCCAGTTGCCTTCTTGGGACTGA	162
	R: TAAGCCTCCGACTTGTGAAGTGGT	
*β*-Actin	F: TGGAA CGGTG AAGGT GACAGC	177
	R:GCTTTTGGGAA GGCAG GGACT	
NF-*κ*B	F: CACTGTCACCTGGAAGCAGAG	139
	R: CACACATCTCCTTTCTCATTGC	
TLR-4	F: TTACAGAAGCTGGTTGCCGT	152
	R: TCCAGGTTGGGCAGGTTAGA	

TLR4: Toll-like receptor 4; NF-*κ*B: nuclear factor-*κ*B; Nrf2: nuclear factor erythroid-derived 2-related factor 2; HO-1: heme oxygenase-1; interleukin-1*β*: IL-1*β*; IL-6: interleukin-6; TNF-*α*: tumor necrosis factor-*α*.

**Table 3:** Effect of dietary Se Y on the growth performance in weaned pigs.

Items	Treatments				*P* value			
	CON	SSY	DT	DSY	SEM	SeY	Diquat	SeY × diquat
1-21								
ADG (g/day)	390.86^a^	335.05^ac^	221.71^b^	271.62^bc^	88.07	0.65	0.01	0.10
ADFI (g/day)	499.42^a^	428.37^ac^	338.86^bc^	377.34^bc^	93.40	0.96	0.02	0.22
F:G (g/g)	1.27^b^	1.27^b^	1.60^a^	1.40^ab^	0.20	0.02	<0.01	0.02

Values are means ± SEM, (*n* = 8), nonchallenged pigs (CON, fed with basal diet), diquat-challenged pigs (DT, fed with basal diet), and SeY-treated pigs (fed with basal diet containing 250 mg/kg SeY) challenged by sterile saline (SSY) or diquat (DSY). ^a,b,c^Mean values within a row with unlike superscript letters were significantly different (*P* < 0.05). ADFI: average daily feed intake; ADG: average daily gain; G/F: the ratio of gain to feed intake.

**Table 4:** Effect of SeY on ATTD of nutrients in weaned pigs.

Items (%)	Treatments				SEM	*P* value
	CON	SSY	DT	DSY		
DM	83.12	83.67	83.65	83.20	0.67	0.89
CP	81.69^b^	86.33^a^	82.14^b^	85.31^ab^	2.57	0.03
EE	73.53	75.27	71.26	81.91	3.47	0.65
Ash	62.18^b^	69.42^a^	63.5^b^	65.76^a^	1.13	0.02
GE	80.98^ab^	86.61^a^	76.96^b^	84.23^a^	1.75	0.04

Values are means ± SEM, (*n* = 8), nonchallenged pigs (CON, fed with basal diet), diquat-challenged pigs (DT, fed with basal diet), and SeY-treated pigs (fed with basal diet containing 250 mg/kg SeY) challenged by sterile saline (SSY) or diquat (DSY). ^a,b,c^Mean values within a row with unlike superscript letters were significantly different (*P* < 0.05). DM: dry matter; CP: crude protein; EE: ether extract; GE: gross energy.

**Table 5:** Effect of dietary SeY on the viscera index in weaned pigs.

Items	Treatments				*P* value			
	CON	SSY	DT	DSY	SEM	SeY	Diquat	SeY × diquat
Liver index	20.35^ab^	20.94^ab^	19.47^bc^	24.90^a^	0.85	0.06	0.32	0.15
Spleen index	5.13	4.75	4.74	5.60	0.74	0.42	0.41	0.10
Kidney index	1.65^ab^	1.81^ac^	1.20^bc^	2.03^a^	0.65	0.09	0.97	0.48
Thymus index	1.04^ab^	1.50^a^	0.74^b^	1.13^ab^	0.10	0.02	0.17	0.47

Values are means ± SEM, (*n* = 8), nonchallenged pigs (CON, fed with basal diet), diquat-challenged pigs (DT, fed with basal diet), and SeY-treated pigs (fed with basal diet containing 250 mg/kg SeY) challenged by sterile saline (SSY) or diquat (DSY). ^a,b,c^Mean values within a row with unlike superscript letters were significantly different (*P* < 0.05).

**Table 6:** Effect of SeY on serum immunoglobulin and cytokine concentrations in weaned pigs upon oxidative stress.

Items	Treatments				*P* value			
	CON	SSY	DT	DSY	SEM	SeY	Diquat	SeY × diquat
TNF-*α* (pg/mL)	466.99^a^	427.6^b^	464.92 ^a^	370.63^b^	15.51	0.03	0.30	0.33
IL-1*β* (ng/L)	42.8^a^	23.12^c^	45.73^a^	34.35^b^	2.43	<0.01	0.03	0.18
IL-6 (ng/mL)	2.46^ac^	1.74^bc^	3.04^a^	1.91^bc^	0.21	0.02	0.33	0.59
TP (gprot/L)	26.31^b^	32.09^a^	24.68^b^	30.38^a^	1.01	<0.01	0.79	0.31
IgG (*μ*g/mL)	306.35^c^	588.03^a^	323.27^c^	497.36^b^	28.74	<0.01	0.09	0.02
IgA (*μ*g/mL)	93.08^b^	166.94^a^	114.28^b^	159^a^	7.90	<0.01	0.37	0.06

Values are means ± SEM, (*n* = 8), nonchallenged pigs (CON, fed with basal diet), diquat-challenged pigs (DT, fed with basal diet), and SeY-treated pigs (fed with basal diet containing 250 mg/kg SeY) challenged by sterile saline (SSY) or diquat (DSY). ^a,b,c^Mean values within a row with unlike superscript letters were significantly different (*P* < 0.05). TP: total protein; IgG: immunoglobulin G; IgA: immunoglobulin A; TNF-*α*: tumor necrosis factor-*α*; IL-6: interleukin-6; IL-1*β*: interleukin-1*β*.

**Table 7:** Effect of SeY on antioxidant capacity of the serum in weaned pig upon oxidative stress.

Items	Treatments					*P* value		
	CON	SSY	DT	DSY	SEM	SeY	Diquat	SeY × diquat
GSH-Px, mg/mL	747.19^b^	878.67^ac^	793.48^bc^	837.24^a^	99.60	<0.01	0.12	0.94
T-AOC, U/mL	0.61^b^	0.57^b^	0.42^b^	1.47^a^	0.13	0.02	0.07	0.02
MDA, nmol/mL	2.49^b^	1.71^c^	3.73^a^	2.06^b^	0.21	<0.01	0.08	0.11
CAT U/mL	2.85^b^	5.85^a^	2.92^b^	5.21^a^	0.47	<0.01	0.71	0.65
SOD U/mL	109.82^b^	129.81^a^	103.29^b^	133.65^a^	3.72	<0.01	0.79	0.31

Values are means ± SEM, (*n* = 8), nonchallenged pigs (CON, fed with basal diet), diquat-challenged pigs (DT, fed with basal diet), and SeY-treated pigs (fed with basal diet containing 250 mg/kg SeY) challenged by sterile saline (SSY) or diquat (DSY). ^a,b,c^Mean values within a row with unlike superscript letters were significantly different (*P* < 0.05). GSH-Px: glutathione peroxidase; T-AOC: total antioxidant capacity, MDA: malondialdehyde; SOD: superoxide dismutase; CAT: catalase.

**Table 8:** Effect of SeY on antioxidant capacity of the liver in weaned pig upon oxidative stress.

Items	Treatments				*P* value			
	CON	SSY	DT	DSY	SEM	SeY	Diquat	SeY × diquat
Liver								
T-AOC, U/mgprot	1.50^ac^	1.30^a^	0.80^bc^	1.33^a^	0.39	0.23	0.02	0.02
MDA, nmol/mL	1.87^a^	1.14^b^	1.78^a^	1.43^ac^	0.47	0.03	0.83	0.40
CAT, U/gprot	72.10^a^	64.80^a^	51.86^b^	72.00^a^	2.87	0.13	0.34	0.03
SOD, U/mL	97.27^a^	93.92^a^	68.63^b^	117.02^a^	5.88	0.02	0.85	0.02
GSH-Px U/mgprot	47.19^c^	58.67^ab^	43.28^bc^	67.24^a^	10.05	0.03	0.05	0.03
Thymus								
T-AOC, U/mgprot	7.05^a^	6.16^a^	4.04^b^	8.71^a^	0.58	<0.01	0.81	0.06
CAT U/mgprot	62.10^a^	64.80^a^	51.86^b^	65.60^a^	2.87	0.03	0.34	0.03
MDA nmol/mL	2.17^b^	1.64^c^	3.78^a^	1.93^bc^	0.57	0.03	0.56	0.30
SOD U/mgprot	97.27^a^	93.92^a^	68.63^b^	107.02^a^	5.88	0.07	0.85	0.02
GSH-Px U/mgprot	80.43^b^	90.56^a^	78.22^b^	98.76^a^	6.44	<0.01	0.67	0.04

Values are means ± SEM, (*n* = 8), nonchallenged pigs (CON, fed with basal diet), diquat-challenged pigs (DT, fed with basal diet), and SeY-treated pigs (fed with basal diet containing 250 mg/kg SeY) challenged by sterile saline (SSY) or diquat (DSY). ^a,b,c^Mean values within a row with unlike superscript letters were significantly different (*P* < 0.05). T-AOC: total antioxidant capacity; CAT: catalase; MDA: malondialdehyde; SOD: superoxide dismutase; GSH-Px: glutathione peroxidase.

## Data Availability

The datasets used to support the findings of this study are available from the corresponding author upon request.

## Additional Points

*Animal Welfare Statement*. All animal care and experimental procedures were performed following the Guide for the Care and Use of Laboratory Animals prepared by the Institutional Animal Care and Use Committee, Sichuan Agricultural University, China.
